# Quinolone prophylaxis for the prevention of BK virus infection in kidney transplantation: study protocol for a randomized controlled trial

**DOI:** 10.1186/1745-6215-14-185

**Published:** 2013-06-21

**Authors:** Atul Humar, John Gill, Olwyn Johnston, Dean Fergusson, Andrew A House, Louise Lebel, Sandra Cockfield, S Joseph Kim, Jeff Zaltzman, Marcelo Cantarovich, Martin Karpinski, Tim Ramsay, Greg A Knoll

**Affiliations:** 1 Department of Medicine, Faculty of Medicine & Dentistry, 2J2.00 WC Mackenzie Health Sciences Centre, University of Alberta, Edmonton, AB T6G 2R7, Canada; 2 Department of Medicine, St Paul’s Hospital, 1081 Burrard Street, Vancouver, BC V6Z 1Y6, Canada; 3 Department of Medicine, Vancouver General Hospital, 855 West 12th Avenue, Vancouver, BC V5Z 1M9, Canada; 4 Clinical Epidemiology Program, Ottawa Hospital Research Institute, 501 Smyth Road, Ottawa, ON K1H 8L6, Canada; 5 Department of Medicine, Western University and London Health Sciences Centre, 339 Windermere Road, London, ON N6A 5A5, Canada; 6 Clinical Epidemiology Program, Ottawa Hospital Research Institute, 501 Smyth Road, Ottawa, ON K1H 8L6, Canada; 7 Department of Medicine, Toronto General Hospital, 200 Elizabeth Street, Toronto, ON M5G 2C4, Canada; 8 Department of Medicine, St Michael’s Hospital, 30 Bond Street, Toronto, ON M5B 1W8, Canada; 9 Department of Medicine, McGill University Health Center, 687 Pine Avenue West, Montreal, QC H3A 1A1, Canada; 10 Department of Medicine, University of Manitoba, 820 Sherbrook Street, Winnipeg, MB R3T 2N2, Canada; 11 Department of Medicine, Ottawa Hospital Research Institute, University of Ottawa, 1967 Riverside Drive, Ottawa, ON K1H 7W9, Canada

**Keywords:** Kidney transplantation, BK virus, Polyomavirus, Quinolone, Levofloxacin, Randomized controlled trial

## Abstract

**Background:**

BK virus infection has emerged as a major complication in kidney transplantation leading to a significant reduction in graft survival. There are currently no proven strategies to prevent or treat BK virus infection. Quinolone antibiotics, such as levofloxacin, have demonstrated activity against BK virus. We hypothesize that administration of a quinolone antibiotic, when given early post-transplantation, will prevent the establishment of BK viral replication in the urine and thus prevent systemic BK virus infection.

**Methods/design:**

The aim of this pilot trial is to assess the efficacy, safety and feasibility of a 3-month course of levofloxacin in the kidney transplant population. This is a multicenter, randomized, double-blind, placebo-controlled trial with two parallel arms conducted in 11 Canadian kidney transplant centers. A total of 154 patients with end-stage renal disease undergoing kidney transplantation will be randomized to receive a 3-month course of levofloxacin or placebo starting in the early post-transplant period. Levofloxacin will be administered at 500 mg po daily with dose adjustments based on kidney function. The primary outcome will be the time to occurrence of BK viruria within the first year post-transplantation. Secondary outcomes include BK viremia, measures of safety (adverse events, resistant infections,*Clostridium difficile*-associated diarrhea), measures of feasibility (proportion of transplanted patients recruited into the trial), proportion of patients adherent to the protocol, patient drop-out and loss to follow-up,and use of quinolone antibiotics outside of the trial protocol.

**Discussion:**

Results from this pilot study will provide vital information to design and conduct a large, multicenter trial to determine if quinolone therapy decreases clinically meaningful outcomes in kidney transplantation. If levofloxacin significantly reduces BK viruria and urine viral loads in kidney transplantation, it will provide important justification to progress to the larger trial. If the full trial shows that levofloxacin significantly reduces BK infection and improves outcomes, its use in kidney transplantation will be strongly endorsed given the lack of proven therapies for this condition.

**Trial registration:**

This trial was funded by the Canadian Institutes of Health Research (grant number:222493) and is registered at ClinicalTrials.gov ( NCT01353339).

## Background

Kidney transplantation is the treatment of choice for end-stage renal disease as it prolongs survival [1], improves quality of life [2] and is less costly when compared to dialysis [3]. Acute rejection, once the major obstacle to successful kidney transplantation, has now been reduced to historically low levels (10% in 2005 [4]). This major advancement in care, however, has been replaced by a new threat, BK virus infection. BK virus is a polyomavirus that occurs worldwide with a prevalence of 60 to 80% in the general population [5]. In kidney transplant recipients, immunosuppression leads to reactivation of the virus. BK virus replication progresses through specific stages: appearing first in the urine (BK viruria), then in the blood (BK viremia) and finally in the kidney transplant as an inflammatory nephritis (BK virus nephropathy) [6]. BK virus nephropathy generally has a poor prognosis with an average transplant failure rate of 46% but reaching as high as 100% in some series [7]. A recent series, however, has shown a more favourable prognosis [8]. In a cohort of patients with definitive BK virus nephropathy, 92% had complete viral clearance with no graft losses at a median follow-up of 34 months [8].

Except for one study published in 1980 [9], there are no randomized controlled trials (RCTs) evaluating strategies to prevent or treat BK viruria, viremia or nephropathy. Based on uncontrolled data, experts [6,10,11] and guidelines [5,7] have recommended that screening be adopted to detect BK viruria or viremia followed by a reduction in immunosuppression. Intuitively a good idea, this approach is problematic since many patients do not actually clear the virus with this strategy [12-14], immunosuppression reduction can lead to acute and chronic rejection [12,15], and, most importantly, long-term outcomes with this screening strategy remain uncertain [16].

We propose to conduct a RCT to determine if we can prevent BK virus infection from occurring rather than trying to treat the virus once replication has been established. Prophylactic strategies such as this are familiar to kidney transplant patients and physicians as they have become the standard of care for the prevention of *Cytomegalovirus* (CMV) [17] and *Pneumocystis* infection [18]. Quinolone antibiotics are safe, commonly used medications [19] that also have antiviral properties against BK virus [20,21]. We hypothesize that the administration of a quinolone, if given early post-transplantation, will prevent BK viral replication in the urine and thus prevent systemic BK virus infection. Preliminary data in kidney transplant recipients have supported this preventive strategy.

## Methods/design

This pilot study is a multicenter, double-blind, RCT comparing a 3-month course of the quinolone antibiotic, levofloxacin, to placebo in 154 kidney transplant recipients. This study has been approved by the Ottawa Hospital Research Ethics Board, Ottawa, ON, Canada (protocol number: 2010292-01H).

### Patient population

The following patients are eligible for this trial: 1) primary or repeat kidney transplant recipients (deceased or living donor); and 2) adults ≥18 years of age.

Exclusion criteria include: 1) patient unable to provide informed consent; 2) greater than 5 days post-transplantation; 3) BK virus nephropathy with a previous transplant; 4) history of allergic reaction to any quinolone antibiotic; 5) history of quinolone-associated tendonitis or tendon rupture; 6) corrected QT interval ≥450 ms; 7) taking medication known to prolong the QT interval, such as class IA antiarrhythmic drugs (for example quinidine, procainamide, disopyramide), class III antiarrhythmic drugs (for example amiodarone, sotalol), azole antifungals (for example fluconazole) or macrolide antibiotics (for example erythromycin); 8) pregnant or breastfeeding, since the safety of levofloxacin in these settings is not established; 9) require quinolone antibiotic for >14 days (for example for UTI prophylaxis); 10) received a multiorgan transplant (for example kidney-pancreas); 11) currently enrolled in another interventional trial; 12) previously enrolled in this study; 13) history of rhabdomyolysis [22]; and 14) significant allergic reactions to ≥3classes of antibiotics, since these patients may have no other option other than quinolones for treatment of common post-transplant infection.

### Trial intervention

The target dose of levofloxacin is 500 mg daily for 3 months. With normal kidney function, a single 500 mg tablet of levofloxacin will produce a maximal urinary drug concentration of 406 mg/l [23]. At a concentration of 250 μg/ml, levofloxacin was shown to inhibit 3/8 BK virus isolates [20]. At a concentration of 500 μg/ml, levofloxacin was able to inhibit the remaining 5/8 isolates [20]. Thus, we expect that the 500 mg tablet of levofloxacin given daily will result in sufficient urinary drug concentration to inhibit BK virus replication. Since this is a proof of concept pilot trial there are no published data to guide the duration of therapy. Given that 80 to 85% of BK virus infections begin within the first 3 months post-transplantation, a 3-month course of levofloxacin should prevent the majority of infections [24,25]. While 2 to 4 weeks of therapy might be the most conservative approach with regards to safety, we believe this would be insufficient to effectively prevent BK virus infection. Similarly, a prolonged course of therapy (for example 6 to 9 months) might prevent more BK virus infections but would have the potential for more adverse events, such as the emergence of resistant organisms. Thus, the 3-month treatment period represents a balance between efficacy and safety appropriate for a pilot study.

Levofloxacin is administered orally once daily in the morning. The 500 mg daily dose is given as two 250 mg capsules to allow for dose reductions if required. At each study visit, creatinine clearance is estimated using the Cockcroft-Gault formula [26] and the dose of levofloxacin is adjusted based on Canadian guidelines [27]. The medication is started as soon as the patient is able to take oral medications but must be started within 5 days post-transplant [28] to ensure early viral replication is prevented. The levofloxacin has been re-encapsulated so that the placebo is identical in appearance to the study medication.

### Other trial maneuvers

Outside of the primary trial intervention, the only major interventions that are being controlled are CMV and *Pneumocystis jirovecii* infection prophylaxis. All participants are receiving prophylaxis against CMV and *Pneumocystis* based on established guidelines [29,30]. Consistent with current clinical practice, patients with a significant unexplained and sustained increase in serum creatinine undergo transplant biopsy. A set protocol has been developed outlining when study medication must be stopped (for example significant *Clostridiumdifficile*-associated diarrhea, rash, tendonitis). Co-interventions that might influence BK infection, such as immunosuppressive medication use, are being thoroughly documented but not controlled. Although immunosuppressive strategies are somewhat variable between centers, mandating a strict immunosuppressive regimen would have limited site participation and generalizability of study findings. More importantly, all immunosuppressive medications have been associated with BK virus infection and the net state of immunosuppression appears more important than individual agents [6,31].

As per our eligibility criteria, the routine use of quinolones for bacterial prophylaxis (for example urinary tract infection (UTI)) is not permitted. The investigators have agreed to not use quinolones for empiric antibiotic therapy. If a quinolone is absolutely necessary, then investigators have this option for the safety of the patient. We expect this situation to be rare given the choices of antibiotics available (for example cephalosporins, amoxicillin/clavulanic acid, nitrofurantoin). A set protocol has been developed to guide the sites on non-study use of quinolones. Once cultures are available, patients are switched to a non-quinolone regimen. If the infection is only quinolone-sensitive or the patient is intolerant/allergic to other antibiotics, then quinolone use (beyond 24 to 48 hours) is allowed. During this time, study medication (placebo or levofloxacin) is withheld. All non-study use of quinolones is being thoroughly documented.

### Protecting against bias

Patients are randomized using a web-based system. A permuted blocked randomization method stratified by center is used to allocate patients. An independent statistician has generated the randomization scheme. The randomization process consists of a computer-generated random listing of the treatment allocations stratified by center in variable permuted blocks of 2 and 4. Only the independent statistician and designated research pharmacist at the coordinating center has knowledge of the randomization codes to ensure concealed randomization of the patients. After screening the patient for eligibility and obtaining informed consent, the study coordinator accesses the trial website and provides the subject’s unique identification,as well as a confirmation of consent and eligibility. The website returns the next available randomization number.

In order to minimize selection and ascertainment biases, physicians, nurses, investigators and research staff are blinded to the randomization schemes and treatments administered. The study medication and placebo are identical in appearance. The designated research pharmacist at the coordinating center is expressly forbidden to discuss individual treatment allocation with the study team or any patients. In addition, the staff at the central laboratory performing the BK virus measurements are not aware of the patient’s treatment allocation. Given that this trial is blinded, contamination and co-interventions should not become imbalanced between the treatment arms. We are documenting the use of co-interventions that may have some impact on BK virus, such as immunosuppressive drug selection, dose and levels [6], leflunomide [6,32], cidofovir [6,33], and intravenous immunoglobulin [6,34].

### Participant follow-up

Patients are being followed for 1 year after the time of randomization. Study visits take place every 4 weeks for the first 24 weeks, then at 32, 40 and 52 weeks. A 1-year follow-up should be sufficient since only 1.0 to 1.3% of patients develop viremia and only 3% develop viruria beyond 40 weeks post-transplantation [24,35].

### Outcome measures

The primary outcome of the pilot study is the time to occurrence of BK viruria within the first year post-transplantation [36]. BK viruria is defined as ≥500 copies/ml of BK virus DNA in the urine, which corresponds to the lower limit of detection for our BK virus assay for urine samples. BK virus infection is determined at each study visit (baseline; every 4 weeks for the first 24 weeks; then at 32, 40 and 52 weeks) by testing for BK viral DNA in urine samples. We have established methods to perform quantitative real-time PCR (qPCR) for the detection of BK virus at our central laboratory at the University of Alberta, Edmonton, AB, Canada. The assay we are using has been in clinical use for the provincial kidney transplant programs and has been used as a reference assay for clinical trials, including one funded by the National Institutes of Health (NIH), Bethesda, MD, USA. Detailed performance characteristics of the assay have been published [37].

Secondary safety outcomes include: 1) incidence and type of all adverse events; 2) incidence of acute rejection; 3) incidence of microbiologically confirmed *C difficile*-associated diarrhea; 4) incidence of other infections (viral, bacterial, fungal) based on established guidelines; 5) incidence of quinolone resistance where a quinolone would have been a therapeutic option (for example *Escherichia coli* UTI); 6) effect of levofloxacin on immunosuppressive drug doses and blood levels [38-40]; and 7) transplant failure and mortality.

Secondary feasibility outcomes include: 1) number of patients transplanted during the recruitment period who are randomized into the trial; 2) proportion of randomized participants who are adherent to the protocol. Participants who take at least 80% of study medication and do not report any episodes of non-adherence will be classified as adherent. Medication use will be measured by pill count at each study visit. In addition, the participants will be questioned about any missed doses. Based on data from the literature [41-44] and the fact that our trial involves 3 months of therapy, we will judge this outcome to be successful if >75% of participants are adherent; 3) use of quinolones outside of the protocol; and 4)proportion of patient drop-out and loss to follow-up.

Secondary clinical outcomes include quantitative BK viral load in urine and the time to occurrence of BK viremia (defined as ≥25 copies/ml of BK virus DNA in the plasma).

### Sample size

Based on data from the literature, we estimate that 35% of patients in the placebo group will develop BK viruria by 1-year post-transplantation [24]. In order to detect an absolute reduction in BK viruria of 20% (from 35 to 15%) with a two-sided alpha error of 0.05, a beta error of 0.2 and a 5% loss to follow-up rate, we would need 154 patients in total (77 per group). The minimal clinically important difference of 20% was justified based on a survey of experts from the Canadian Renal Transplant Study Group. The investigators wanted to see a substantial effect on viruria in order to justify proceeding with a larger trial examining the more clinically relevant endpoint of time to doubling serum creatinine, transplant failure or death.

### Statistical analysis

Baseline characteristics of patients in the two treatment arms will be assessed using frequency distributions and univariate descriptive statistics, including measures of central tendency and dispersion. Analyses will be performed by intention-to-treat. Since this is a pilot study to assess biologic efficacy, the intention-to-treat analysis will be supplemented by a sensitivity analysis that excludes patients who did not complete the allocated treatment plan. The primary analysis will use a nonparametric log-rank test, stratified by center, to compare the time to occurrence of BK viruria between the control and levofloxacin treatment groups. Kaplan-Meier survival curves will also be plotted to visually assess differences in incidence over time.

For secondary safety outcomes, the proportion of adverse events occurring in each treatment arm will be compared using an unadjusted chi-square test or Fisher’s exact test if cell sizes are small. We will evaluate overall adverse events as well as specific serious adverse events (for example *C difficile*-associated diarrhea). For the secondary feasibility outcome of recruitment, we will calculate the proportion (and 95% confidence interval) of all patients transplanted during the recruitment period who are randomized into the trial. In addition, we will calculate the proportion of patients that are eligible but consent declined, eligible but not approached and not eligible. These analyses will be performed at each site and for the trial overall. For the secondary feasibility outcomes of adherence, non-study quinolone use, drop-out and loss to follow-up, the proportion of each outcome will be compared between the arms with an unadjusted chi-square or Fisher’s exact test. Since this is a pilot study, we have not planned for any formal interim analyses.

## Discussion

BK virus infection has emerged as a major complication in kidney transplantation. There are no proven strategies to prevent or treat BK virus infection. This trial will assess the efficacy, safety and feasibility of a 3-month course of levofloxacin to prevent BK virus infection following kidney transplantation. While there are concerns about possible antimicrobial resistance and complications, such as *C difficile*-associated diarrhea, we are closely monitoring safety and adverse events as part of this trial. Results from this pilot study will provide vital information to design and conduct a large, multicenter trial to determine if levofloxacin therapy decreases major clinical outcomes, such as biopsy-proven BK nephropathy or graft loss in kidney transplantation. If levofloxacin significantly reduces BK viruria and urine viral loads in kidney transplantation, it will provide strong support of biologic effect and justification to progress to the larger trial. If the full trial shows that levofloxacin significantly reduces BK infection and improves outcomes, the use of this preventive therapy will be a major innovation in the management of kidney transplant recipients, especially given the lack of proven therapies for this condition.

## Trial status

The first participant was randomized in December 2011 and recruitment is ongoing as of 28 May 2013.

## Abbreviations

CMV: *Cytomegalovirus*; NIH: National Institutes of Health; PCR: Polymerase chain reaction; qPCR: Quantitative real-time PCR; RCT: Randomized controlled trial; UTI: Urinary tract infection.

## Competing interests

The authors declare that they have no competing interests related to this study.

## Authors’ contributions

AH, JG and GAK conceived the study, participated in its design and submission to the Canadian Institutes of Health Research for funding, and drafted the manuscript. DF, AH, SC, MC, MK and TR participated in the design of the study and submission to the Canadian Institutes of Health Research for funding. OJ, SJK, LL and JZ contributed to the design of the study. All authors read and approved the final manuscript.
